# Language-to-music transfer effects depend on the tone language: Akan vs. East Asian tone languages

**DOI:** 10.3758/s13421-023-01416-4

**Published:** 2023-04-13

**Authors:** Sarah C. Creel, Michael Obiri-Yeboah, Sharon Rose

**Affiliations:** 1 UC San Diego Cognitive Science, 9500 Gilman Drive Mail Code 0515, La Jolla, CA 92093-0515 USA; 2 Georgetown University Linguistics, Washington, DC USA; 3 UC San Diego Linguistics, San Diego, CA USA

**Keywords:** Music cognition, Auditory perception, Cross-cultural research, Transfer effects, Tone languages

## Abstract

Recent research suggests that speaking a tone language confers benefits in processing pitch in nonlinguistic contexts such as music. This research largely compares speakers of nontone European languages (English, French) with speakers of tone languages in East Asia (Mandarin, Cantonese, Vietnamese, Thai). However, tone languages exist on multiple continents—notably, languages indigenous to Africa and the Americas. With one exception (Bradley, *Psychomusicology*, *26*(4), 337–345, 2016), no research has assessed whether these tone languages also confer pitch processing advantages. Two studies presented a melody change detection task, using quasirandom note sequences drawn from Western major scale tone probabilities. Listeners were speakers of Akan, a tone language of Ghana, plus speakers from previously tested populations (nontone language speakers and East Asian tone language speakers). In both cases, East Asian tone language speakers showed the strongest musical pitch processing, but Akan speakers did not exceed nontone speakers, despite comparable or better instrument change detection. Results suggest more nuanced effects of tone languages on pitch processing. Greater numbers of tones, presence of contour tones in a language’s tone inventory, or possibly greater functional load of tone may be more likely to confer pitch processing benefits than mere presence of tone contrasts.

## Introduction

Tone languages are those that use pitch patterns to convey differences in meaning at the word level. Of interest to cognitive scientists, several previous studies report that listeners who speak tone languages show benefits in musical pitch perception over those who do not speak tone languages (e.g., Burnham et al., 2014; Hutka et al., 2015; Wong et al., 2012), including children (Creel et al., 2018; Deroche et al., 2019). These findings are consistent with transfer of pitch processing facility from spoken language to nonspeech stimuli, implying that language processing is integrated with other domains of cognition rather than an isolated, modular ability. However, existing findings of language-to-music transfer have some limitations. In particular, nearly all such studies have examined tone languages and listeners who originate from East Asia. Such tone languages (e.g., Mandarin, Thai, Cantonese) share several properties, including possessing four or more distinct tones, many of which are contour tones. However, tone also abounds in African languages (Kutsch Lojenga, 2018; Odden, 1995, 2020), indigenous languages of the Americas (Caballero & Gordon, 2021; DiCanio & Bennett, 2021), and some Papuan languages (Cahill, 2011; McPherson & Dryer, 2021). Such languages may deploy tone somewhat differently, including systems with level or register tones and different uses of tone in conveying grammatical meaning. Examining pitch processing benefits in non-Asian languages widens the scope of inquiry and can fine-tune understanding of the relationship between a language’s tone properties and the likelihood of transfer. To address this empirical gap, in the current study, we test pitch perception in speakers of the tone language Akan, a Niger-Congo Kwa language spoken in Ghana.

### How pitch is used in different languages

In trying to understand potential roles of specific tone language systems on nonspeech pitch perception, it is important to understand how pitch is used in different languages. Pitch—that is, the psychoacoustic correlate of the fundamental frequency of a signal—can pattern in consistent ways within a language, but the nature of the consistency depends on the language. Patterns in pitch are relevant in a range of languages at multiple levels of structure: for all languages, at the level of the phrase, and for tone languages, at the level of a single syllable or word. Many so-called intonation languages—or as we call them here, nontone languages—use phrase-level pitch patterns to differentiate sentence types or convey focus. This includes languages such as English, French, and Spanish. In English, for example, a statement (*She’s here* with falling final pitch) can be distinguished from a declarative question (*She’s here?* with rising final pitch) by intonation.

Other languages, referred to as *tone languages*, additionally use pitch features to distinguish word meanings (in addition to intonational functions of pitch; see, e.g., Downing & Rialland, 2017; Liu & Xu, 2005; Xu, 1999; Yuan et al., 2002; for the complex interplay between tone, intonation, and focus). A classic example from Mandarin is that *ma* has four different meanings depending on its fundamental frequency (f0) pattern (Tone 1 high level tone: “mother”; Tone 2 rising tone: “hemp”; Tone 3 fall-rise: “horse”; and Tone 4 fall: “scold”). Maddieson (2013) suggests that 42% of the world’s languages use tone, based on a sample of 527 languages. Of the 42%, Maddieson classifies 60% as “simple” 2-tone systems, including pitch-accent languages such as Norwegian, with the remaining 40% being “complex” tone systems (three or more tones). Maddieson further acknowledges that this estimate of tone language prevalence may be an underestimate due to uneven sampling density in the source data. In any case, despite the widespread presence of tone as a linguistic feature, the most-studied tone languages in the psycholinguistics literature are those of East Asia, including Mandarin, Cantonese, Thai, and Vietnamese. However, many other languages from disparate language phyla also use tone, including hundreds of African languages (e.g., Akan, Yoruba, Dinka, Shona, Nama), many indigenous American languages (e.g., Navajo, Chatino, Itunyoso Trique, Ticuna), and some Papuan and Oceanic Languages (e.g., Skou, Awa).

Of further interest, not all tone languages use pitch in the same ways, and it is possible that different types of tone or uses of tone may have different effects on pitch perception. First, at a high level of description, languages are often classified as having *register* (or level) *tones* versus *contour tones* (Pike, 1948). Register tones maintain relatively level pitch across the course of the tone (many African tone languages), while contour tones change pitch systematically over the course of the tone (many East Asian tone languages). This classification is not strict: Languages with contour tones also have level tones (e.g., Tone 1 in Mandarin is a level high tone), and register tone languages may have contour tones either as primitives or derived from the fusion of two level tones. Second, to varying degrees, tone languages use *other phonetic cues* that correlate with pitch patterns, such as vowel duration and voice quality (Andruski & Ratliff, 2000; Garellek et al., 2013) as well as amplitude (see, e.g., Liu & Samuel, 2004; Zhang et al., 2022). For example, in Mandarin, *ma* is longer when it has Tone 3 versus Tone 4 (Whalen & Xu, 1992), and Mandarin Tone 3 correlates with creaky voice quality (Kuang, 2017). In some cases these other cues may be more critical than pitch to identifying the sound category (Kuang, 2013). Languages also vary in *number of tones*: two (Akan), three (Yoruba), four (Mandarin), six (Cantonese), nine (Dong), while others have been characterized as having up to 14 distinct tones (Wobé [Bearth & Link, 1980] or Santa Lucía Teotepec Chatino [McIntosh, 2015]). Finally, some tone languages, including East Asian tone languages, use tone to differentiate *lexical meanings* (e.g., mother vs. horse), while others, including many African and American indigenous tone languages, use tone for both lexical and *grammatical purposes*. For example, in Akan, tense/aspect/mood categories are distinguished by tone (Dolphyne, 1988; Paster, 2010). As one illustration, bìsá, with a low-high pitch pattern, means “ask (habitual),” while bìsà, with a low-low pattern, means “ask (imperative)!,” showing grammatical tone.

Note that a level contrastive tone does not mean that that tone has entirely stable pitch. Indeed, the f0 of a tone may dip or rise at the edges depending on the surrounding tones, consonant influence, or position in the sentence. This may give rise to a surface phonetic contour. In some languages, such edge tone effects can become phonologized. For example, in Yoruba, a sequence of high low /H L/ phonological tone is realized as a high falling sequence [H H͡L] as the high tone extends or spreads into the following syllable which hosts the L, creating a fall (H͡L) (Connell & Ladd, 1990). The reverse sequence of L H shows the same effect, whereby L spreads onto the following H, creating a rising tone [L L͡H]. The contour tones are not part of the basic tone inventory of Yoruba, as they are derived and predictable in their distribution, but they do form part of the phonological system. Likewise, a contour contrastive tone will generally maintain its fall or rise shape, although this, too can also be affected by context. For example, a contour tone may require a syllable to have a particular length or be in a prominent position for the contour to be fully realized (Zhang, 2004).

It is not clear from perceptual confusability studies whether level tones or contour tones as described at either phonological or phonetic levels render a tone system “harder” from a perceptual point of view. There is evidence for confusability both within and across these tone types (e.g., Khouw & Ciocca, 2007; Wayland & Guion, 2003; Whalen & Xu, 1992; Zsiga & Nitisaroj, 2007). However, a larger number of tones affords more possibilities for tone confusions, such that one might expect more precise tone realizations in languages with more tones, which might in turn lead speakers of those languages toward finer pitch perception acuity, at least in language (whether or not it generalizes to nonlinguistic pitch; see Patel, 2011, discussed below). In dispersion theory (Lindblom, 1986), vowels are distributed in acoustic space based on maximal perceptual distinctiveness. When the concept is applied to tone, the size of the tonal space across languages remains relatively fixed despite the number of tones (Alexander, 2010); moreover, if the number of level tone contrasts in a language exceeds four, voice quality differences are employed (Kuang, 2013). It is not clear how tone dispersion might apply to pitch acuity within a fixed tonal space, but Hu et al. (2020) recently reported that speakers of Dong, a Chinese language with nine tones, show better pitch acuity than speakers of Mandarin (four tones only). Relatedly, Yang (2019) showed that Thai (five tones) learners of Mandarin were better at tone production than similarly experienced Yoruba (three tones) learners of Mandarin, though Yang’s study leaves open what degree of this finding is attributable to the contour/level distinction vs. the different numbers of tones. Three of the Thai tones have been analyzed as register tones phonologically (Burnham et al., 2014; Zsiga & Nitisaroj, 2007), even if they are phonetically produced with slight rise or fall.

### Tone languages and pitch processing﻿

The basis of the tone-language transfer hypothesis is the observation that tone-language speakers as a group excel at pitch perception tasks relative to nontone-language speakers. Perhaps not surprisingly, these pitch perception benefits appear in linguistic stimuli (Bent et al., 2006; Choi et al., 2019). In fact, Choi et al. (2019) report that Mandarin speakers use pitch cues to lexical stress in English more accurately than English speakers do, suggesting transfer of pitch processing from one’s native language to another language (though English speakers use cues to stress besides pitch; e.g., Chrabaszcz et al., 2014).

More interestingly for present purposes are findings that greater pitch sensitivity in tone language speakers also show up in *nonspeech* (musical or nonspeech auditory) materials, suggesting transfer from language to music. Amongst young adults, tone-language speakers show better pitch processing than nontone-language (usually English-speaking) control listeners (Cantonese: Bidelman et al., 2013; Hutka et al., 2015; Wong et al., 2012; Mandarin: Bradley, 2016; Giuliano et al., 2011; Hove et al., 2010; Jasmin et al., 2021; Pfordresher & Brown, 2009; Wong et al., 2012; Thai: Burnham et al., 2014; Yoruba: Bradley, 2016), and speakers of Dong, a nine-tone language, show better pitch discrimination than speakers of Mandarin, a four-tone language (Hu et al., 2020). This tone advantage is evident as early as 4 years of age (Creel et al., 2018; see also Deroche et al., 2019; both compared Mandarin-speaking vs. English-speaking children). Finally, a recent large-*N* preprint study by J. Liu et al. (2021), which included thousands of speakers each of Cantonese, Hokkien, Mandarin, Thai, and Vietnamese, as well as thousands of nontone language speakers, reported an advantage for melody change detection in tone speakers.

There have been some failures to replicate tone-language advantages in pitch perception (Bent et al., 2006; Bidelman et al., 2011; Nan et al., 2010; Peretz et al., 2011, 2013; Stagray & Downs, 1993). Several of those studies tested very fine pitch gradations that were smaller than musically relevant differences (Bidelman et al., 2011; Peretz et al., 2011; Stagray & Downs, 1993). Others found perceptual distortions that were argued to result from details of tone use in the tone speakers’ languages (Bent et al., 2006; Peretz et al., 2011; Stagray & Downs, 1993). Another study did find some pitch processing advantages in tone-speaking over nontone-speaking 6-year-olds (Peretz et al., 2013) but argued against these as evidence of a tone language advantage, in part because tone speakers also excelled in a rhythm task (implying overall better test performance, not a pitch-specific advantage). However, on close examination of Peretz et al.’s (2013) Table 2, it appears that tone-language 6-year-olds’ rhythm test scores are significantly *lower*, not higher, than nontone speakers’, which would actually bolster evidence for a pitch-specific advantage. Interestingly, J. Liu et al.’s (2021) large-*N* study which reported melody change detection advantages for tone speakers also reported a tone-language *dis*advantage for fine-grained pitch and beat-processing tasks. Thus, the preponderance of results are consistent with a tone-language advantage in musically relevant pitch perception, but one which may not extend to fine-grained pitch differences.

Strikingly, almost all studies in which tone languages show pitch perception advantages involve East Asian tone languages with four or more tones. The major exception is Bradley (2016), who tested 15 speakers of Yoruba—a language with three level tones spoken in Nigeria—in a melody change detection task, plus 26 Mandarin speakers and 26 English speakers. Mandarin and Yoruba speakers exceeded English speakers at detecting changes in *musical pitch contour* (e.g., a pattern such as CEDF vs. a pattern such as CDEF in which two notes are interchanged) and more-challenging changes in exact *musical pitch interval* (e.g., a pattern such as ACEG vs. a pattern such as ACFG or ACDG in which one note is higher or lower without changing contour). This is, to our knowledge, the only evidence of pitch processing transfer to music from a tone language outside East Asia, or from a language with less than four tones. These results suggest that transfer occurs for both East Asian and African tone language speakers, and that tone number does not result in differences in musical pitch processing benefits, at least with respect to interval and contour dimensions. However, this is only one study on a single African language, and Yoruba has been reported to have derived surface contour tones (Connell & Ladd, 1990), which could make it more tonally complex than its three-tone description would imply.

### Evidence of music to language transfer

Also relevant is evidence of transfer in the opposite direction, from music to language. Patel’s (2011) OPERA hypothesis posits that the pitch acuity required in music performance trains the brain toward higher-acuity linguistic pitch processing. Consistent with OPERA, musicians (compared with nonmusicians) show greater brain sensitivity to pitch changes in spoken sentences in familiar and unfamiliar languages (Deguchi et al., 2012; Kraus et al., 2014; Marques et al., 2007; Parbery-Clark et al., 2009; Schön et al., 2004; Wong et al., 2007), and they detect linguistic pitch changes better (Bowles et al., 2016; Cooper & Wang, 2012; Delogu et al., 2006, 2010; Marie et al., 2011; Wong & Perrachione, 2007). While most such studies are correlational due to the difficulty of doing long-term interventions (though see Chobert et al., 2014; Kraus et al., 2014), perceptual permeability from music to language lends plausibility to perceptual permeability from language to music.

### Limitations of the existing literature

While existing research suggests that speaking a tone language benefits pitch perception, there are some limitations in this literature. First and foremost, tone languages tested are almost entirely tone languages of East Asia (except for Bradley, 2016). Second, a methodological concern is that task controls are sometimes but not always included. This raises the possibility that apparent benefits of tone languages are in fact some more general positive effect on task performance, and not increased pitch acuity specifically.

### The current study

Here, we compare three groups of speakers, two of which have figured prominently in the tone language transfer literature (English speakers and East Asian tone language speakers), and one which has not (Akan speakers). Akan has several dialects including Asante Twi, Akuapem, Akyem and Fante; the term Twi is often used to refer to Akan in general. Akan has two level tones (Dolphyne, 1988; Genzel, 2013), as well as an additional process called “downstep”—that is, the lowering of the second of a sequence of high tones within a word or sentence. This produces three surface pitch levels (Abakah, 2000; Genzel, 2013). As described earlier, tone differences in Akan are often used to convey differences in grammatical function (Paster, 2010). Another possibly relevant piece of information is that Akan culture has a history of musical surrogate language systems (McPherson, 2018; Sicoli, 2016), involving *atumpan* (barrel or “talking” drums), *mmɛntia & seseɛ* (animal tusk trumpets), *seperewa* (harp lute), and the double bell (Nketia, 1963). These systems convey the pitch of the language via the musical instruments, suggesting possible transfer of tone awareness outside of language to music.

In the two experiments described here, we presented all three groups with two types of musical perceptual discrimination (same–different) questions. To assess listeners’ detection of pitch changes, we presented pairs of melodies that differed only in *one pitch*. As a control to assess general adherence to instructions and understanding of the task, we presented pairs of melodies that contained identical fundamental frequencies but differed in the *instrument* that was playing (see Creel, 2014; Creel et al., 2018; Hutka et al., 2015, for similar control tasks). Experiment 1 presented both types of changes in intermixed order. Experiment 2 presented the two types of changes in separate blocks, to address the concern that results of Experiment 1 were affected by different listeners interpreting the task differently.

## Experiment 1

In this experiment, we compared three groups of listeners: Akan speakers (who also speak English); nontone language speakers (who speak English); East Asian tone language speakers (who also speak English). The latter two groups are those often compared in studies reporting tone language benefits in pitch perception, and serve as an internal replication of previously reported effects: we predict that East Asian tone language speakers will outperform nontone language speakers in melody change detection. The most interesting outcome concerns speakers of Akan. If the tone language benefit in pitch perception extends to Akan speakers, then they too should outperform English (nontone language) speakers in melody change detection. However, if the tone language benefit does not extend to Akan, then Akan speakers should not outperform English speakers in melody change detection. A further prediction might be that, if number (or type) of tones matters on top of the tone/nontone distinction, East Asian tone speakers (four or more tones, including contours) may outperform Akan tone speakers (two level tones). We do not expect groups to differ in instrument change detection performance. Thus, we examine the Trial Type (melody change, instrument change) × Group interaction.

### Method

#### Participants

Group differences in melody change detection, accompanied or unaccompanied by overall group performance differences (melody and instrument change detection combined), should manifest as a two-way interaction in a Language Group × Trial Type analysis. Power analyses were conducted using G*Power (Faul et al., 2007). Assuming effect size *f* = .25 and a correlation = 0 between measures (a conservative assumption), we needed 27 participants per group to achieve power ≥.80 for the interaction. To include our counterbalancing constraints, we aimed to recruit 40 participants per group.

We tested 41 Akan speakers (15 female, 25 male, one did not state) from a pool of college student participants at the University of Education, Winneba in Ghana. All reported speaking Akan dialects including Twi (35), Fante (3), and one each Akuapem, Nafara, and Sefwi. As comparison, and to address previous research on tone language advantages, we also tested a set of 83 listeners in the US who were students at UC San Diego, about half of whom spoke an East Asian tone language (enumerated below), and half of whom did not. Due to lack of clarity on the presence versus absence of tone advantages in certain language types, we removed 14 participants based on reported language knowledge: speakers of pitch accent languages (four Japanese, two Swedish) and languages that may be undergoing tonogenesis (Korean; six speakers—see Silva 2006; Kang & Han, 2013).We also removed a speaker of Burmese from the tone language group, as Burmese tone covaries with substantial voice quality differences (Gruber, 2011). The final sample included 35 nontone speakers (22 female, 12 male, one did not state). Of these, most (32) reported being dominant in English, two in Farsi (English as second language), one in Spanish (English as second language). Of the 32 English-dominant participants, 29 reported knowledge of other languages, mostly Spanish (24) as well as Tagalog (2) and one each of Farsi, Hebrew, and sign language (presumably American Sign Language). As none of these additional languages are tone languages, and most participants were predominantly English speakers, we refer to this group as the English group for convenience. We also retained 33 East-Asian tone language speakers (22 female, 11 male), who reported speaking Chinese (dialect unspecified but likely Mandarin; 10), Mandarin (10), Cantonese (7), Vietnamese (5), and Toishanese (1). In terms of tone inventories, Mandarin has four tones (one level and three contour), Cantonese has six tones (three level and three contour; Bauer & Benedict, 1997), Vietnamese has five or six tones depending on dialect (Hanoi [one level, five contour; Brunelle, 2011; Kirby, 2011]; Ho Chi Minh [one level, four contour; Brunelle, 2011]), while Toishanese has five tones (three level, two contour; Cheng, 1973).

#### Stimuli

Stimuli consisted of 12 six-note melodies whose notes were drawn from the Western major scale. Across all melodies, frequencies of occurrence of scale tones matched those of Youngblood’s (1958) count of scale tones in classical Western music. Essentially, the tonic (first scale degree) and dominant (fifth scale degree) tones were the most frequent, followed by the second and third scale degrees, then the remaining scale tones, a characteristic pattern in Western and Western-related music (see Krumhansl, 1990). Twelve original melodies were created using these constraints, such as A_3_C_4_E_4_C_4_D_4_G_4_.^1^ (Note that this constraint was imposed only at the single-note level, not note-to-note transitions, limiting the extent of their resemblance to real Western music.) Next, for each melody, another melody with a single changed note was created, such as A_3_C_4_**D**_4_C_4_D_4_G_4_. The changed note was also a note in the scale, differed only in 1-2 semitones from the note it replaced, and did not change the local pitch contour of the original melody. Contour-preserving changes and key-preserving changes are more challenging to detect and thus represent a more stringent test of pitch change detection (see, e.g., Bradley, 2016; Dowling, 1991). Our approach was modeled on a task from Bidelman et al. (2013), who used six-note melodies that had altered versions where one note was mistuned by 0.5 semitones. Initial stimulus generation suggested that 0.5-semitone deviations were quite challenging to distinguish, so we used a larger pitch deviation. Half of the note changes each moved the original note downward, half upward. Half were a semitone change, half a whole tone change. Changes were equally likely to occur on the second, third, fourth, or fifth note in the sequence. There were no changes on initial or final notes, which are easier to detect.

After composition, each melody was transposed to one of 12 different major keys so that key was consistent within a trial but randomly varied from trial to trial, diminishing the effect of preceding context on pitch change detection. In order to test for instrument change detection, all melodies were synthesized as sequences of quarter notes in Finale (2009; MakeMusic, Inc.) at a tempo of 185 beats per minute (324 ms/note). Four different instrument sounds were used: muted trumpet, vibraphone, bassoon, and alto saxophone. These instrument sounds have previously been shown to vary in perceptual similarity, such that the first two are quite distant while the last two are fairly similar (Iverson & Krumhansl, 1993), providing some variation in difficulty. All melodies occurred in all instruments.

#### Procedure

The experiment was programmed in PsychoPy2 (Version 1.90.1; Peirce et al., 2019). Viewers read instructions in Akan (Ghana) or in English (USA) and responded by pressing one of two keys on a keyboard. On each trial, they heard two six-note melodies with a 500-millisecond pause in between. They were asked to respond “same” if the two melodies were completely the same, and “different” if they were different. Six example trials with accuracy feedback preceded the main task, three same, three different, in the same order for all participants. Of the three “different” trials, one differed in four notes (and overall contour), another differed in three notes (and overall contour), and the other differed in musical instrument (saxophone vs. bassoon). Following the example trials, participants completed 72 experimental trials: 24 same, 24 different-melody, and 24 different-instrument (half distinct-sounding, half similar-sounding). All participants completed both the current experiment and an unrelated vowel perception experiment, half before and half after the current experiment. Following the main experimental tasks, all subjects completed a questionnaire detailing language use and music experience.

### Results

**Fig. 1:**
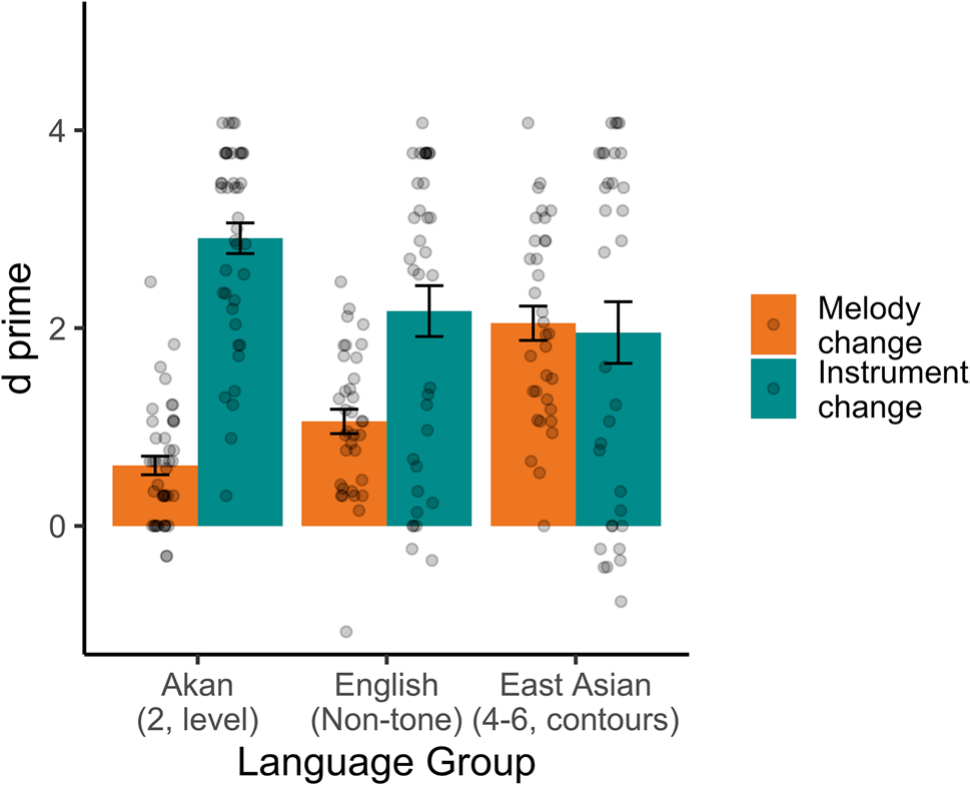
Experiment 1, *d*-primes to melody change versus instrument change trials across language groups, with standard errors. Points are individual participants

Hits (correct responses on different trials) and false alarms (incorrect “different” responses on same trials) were used to calculate *d*-prime (*d'*) scores (Fig. 1), a bias-free estimate of change detection. For scores of 0 or 1, which would produce values of ±infinity when computing the *z*-score components of *d'*, a value of 1/24/2 ≈ .021—corresponding to half of one test item—was added or subtracted to pull the score away from floor or ceiling, respectively. We then compared *d'* across the three groups in a two-way 2 × 3 mixed-design analysis of variance (ANOVA), with trial type (melody-change, instrument-change) as the within-subject predictor and language group (English [nontone] speakers, Akan speakers, East Asian tone speakers) as the between-groups predictor. If there is a tone language advantage, then Akan speakers and East Asian tone speakers should outperform nontone English speakers on melody-change trials, with instrument trials as a baseline.

There was an effect of trial type, *F*(1, 106) = 52.95, *p* < .0001, such that instrument-change trials were overall better detected. There was no overall effect of language group, *F*(2, 106) = 2.13, *p* = .12, suggesting similar overall detection rates across groups. Trial type and language group interacted, *F*(2, 106) = 17.88, *p* < .0001, indicating differences in performance across groups in the two different trial types. To test the nature of the interaction, we computed *t* tests for each group, with Bonferroni correction on *p* values. These indicated that the differences were not the ones predicted: overall, instrument trials were better detected, but the speakers who showed the largest difference between instrument and pitch trials were Akan speakers, *t*(40) = 11.13, *p*_*B*_ < .0001, followed by English speakers, *t*(34) = 3.68, *p*_*B*_ = .002, and, finally, East Asian speakers, *t*(32) = 0.27, *p*_*B*_ = 1.0, who showed a numerically reversed pattern (pitch > instrument). To examine this further, we compared each pair of groups to each other in 2 × 2 ANOVAs, again Bonferroni corrected. This allowed us to compare each pair of groups with instrument-change trials as a baseline. If we think of the melody − instrument difference as a “melody disadvantage” score, both Akan and English speakers had a melody disadvantage, but it was larger for Akan speakers than English speakers, *F*(1, 74) = 10.88, *p*_*B*_ = .004, and larger for Akan speakers than East Asian speakers, *F*(1, 72) = 40.01, *p*_*B*_ < .0001. This is inconsistent with a pitch perception advantage for Akan speakers. Still, consistent with an East Asian tone language advantage for pitch perception, English speakers undershot East Asian speakers, *F*(1, 66) = 6.81, *p*_*B*_ = .03.

#### Exploratory analyses: Music experience

**Fig. 2:**
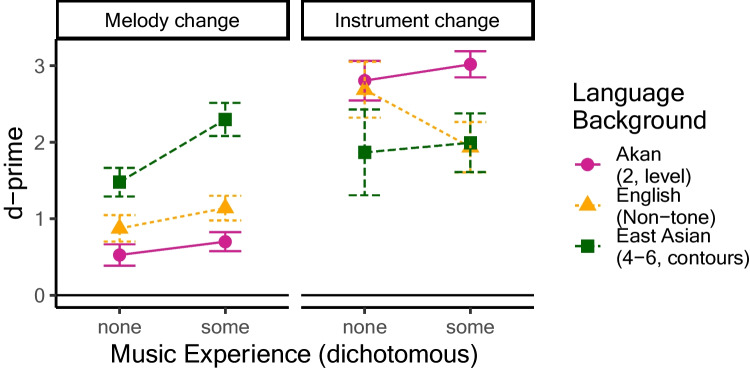
Experiment 1, *d*-prime values, split by absence versus presence of music experience. Melody change detection is stronger with musical experience, but music effects do not explain the effects of language group

Results do not appear to be obscured by major confounds of music experience with language group. An ANOVA on years of experience playing an instrument and/or singing, with language group as a predictor, did not show cross-group differences, *F*(2, 106) = 0.99, *p* = .37. Another way to examine this is to compare results stratified by amount of music experience, either none at all (0 years playing or singing) or more than none (Fig. 2; see also Table 1). This binary grouping is useful given the large number of zero values present in each group. Since this factor was not evenly distributed across participants or groups, and R computes sums of squares sequentially, ANOVAs were run twice: once with group as the first between-groups factor, allowing analysis of music experience (and its interactions) with group variance (and its interactions) removed; and once with music experience as the first between-groups factor, allowing assessment of group variance with music experience variance removed.

**Table 1:** Music experience in each group

Experiment	Language background	Music experience (*n*)		Mean music years (*SD*)
		Yes	No	
Experiment 1	Akan (2, level)	20	21	4.02 (0.76)
	English (nontone)	24	11	5.26 (0.95)
	East Asian (4–6, contours)	23	10	5.64 (0.87)
Experiment 2	Akan (2, level)	14	24	3.97 (0.02)
	English (nontone)	29	8	6.49 (0.10)
	East Asian (4–6, contours)	30	9	8.31 (0.98)

An ANOVA with music experience (present, absent), language group, and trial type as factors did not yield effects, *F*(1, 103) = 0.79, *p* = .38, or interactions with music experience—with group: *F*(2, 103) = 1.57, *p* = .21; with trial type: *F*(1, 103) = 2.03, *p* = .16; with Group × Trial Type: *F*(2, 103) = 0.90, *p* = .41. To fully explore possible influences of musical experience, however, we examined findings for melody-change scores and instrument-change scores separately. Melody-change trials were affected by language group, *F*(2, 103) = 29.90, *p* < .0001, and music experience, *F*(1, 103) = 6.34, *p* = .01, suggesting that more musically experienced listeners tended to be better at detecting melody changes, but there was no interaction, *F*(2, 103) = 1.64, *p* = .20. Thus, group differences in melody change detection appear robust to amount of musical experience. Instrument-change trials were affected by language group, *F*(2, 103) = 4.13, *p* = .02, but not music experience, no main effect, *F*(1, 103) = 0.13, *p* = .72; or interaction; *F*(2, 103) = 1.09, *p* = .34.

#### Exploratory analyses: Do individual participants pick individual strategies?

**Fig. 3:**
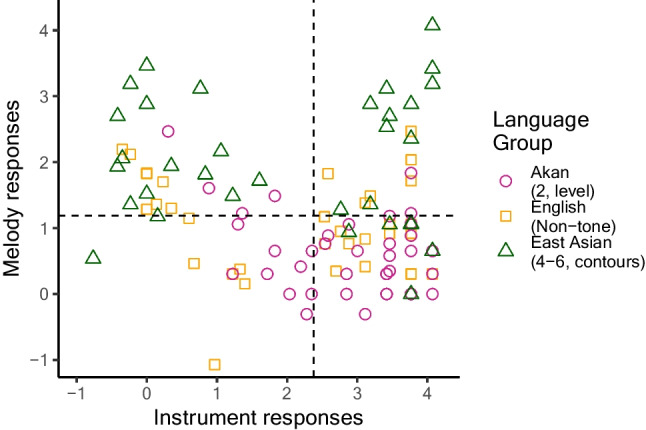
Experiment 1 response patterns. Many participants responded mainly to instrument change (lower right) or mainly to pitch change (upper left), but not both. Filled shapes indicate >0 years music experience. Dashed lines indicate grand means in each condition

One question that arose was the extent to which results are driven by task interpretation, rather than actual pitch sensitivity. Figure 3 (see also Table 2) shows instrument change detection on the *x*-axis, and melody change detection on the *y*-axis. Akan speakers were predominantly instrument responders (lower right); nontone speakers tended to be instrument responders; and East Asian tone speakers had more melody responders (upper left) and dual responders (upper right) than either other group.

**Table 2:** Percentages of each group (*n*) above versus below mean *d'* for melody changes and instrument changes

	Akan tone	US nontone	US East Asian tone
Instrument above: Instrument responder	63% (26)	43% (15)	18% (6)
Melody above: Melody responder	10% (4)	23% (8)	42% (14)
Both above: Dual responder	5% (2)	17% (6)	33% (11)
Neither above: Low sensitivity	22% (9)	17% (6)	6% (2)

This pattern of results complicates interpretation somewhat. Are listeners really perceiving differently as a function of language background, or do members of the different groups tend to interpret the task differently? Perhaps listeners who are less steeped in standardized tests with tricky response options may listen for salient differences—instruments—and report those, while standardized-test veterans may think the instrument trials are too obvious and therefore a trick and scrutinize for small differences. The Ghanaian educational system has two high-stakes national examinations: the Basic Education Certificate Examination for elementary students and the West African Senior Secondary Certificate Examination for secondary students, which employ multiple-choice and free-response questions (Ajayi, 2014; Owusu, 2021). Classroom tests are also employed, but Quansah et al. (2019) reports that Ghanaian high school teachers have limited test construction skills, and tests vary widely in their content, style and effectiveness. The employment of standardized tests is otherwise not as widespread in Ghana as in the U.S., where students take 112 examinations from kindergarten through Grade 12 (Hart et al., 2015), or China, where the *gaokao* serves as the nationwide college entrance exam and is the subject of yearslong student preparation (Larmer, 2014). In the postexperiment comments, four participants each in the nontone and East Asian tone speakers—but none of the Akan speakers—commented that they thought the purpose of the study had to do with “small changes” or “subtle differences” in melodies. A strategy of monitoring for minute differences is substantiated by a higher false-alarm rate (saying “different” on same trials) in the two U.S. groups than the Akan group. Akan listeners made only 1.5% false alarms, lower than English listeners at 7.2%, *t*(74) = 5.02, *p* < .0001, and East-Asian tone listeners at 6.8%, *t*(72) = 4.68, *p* < .0001. East-Asian tone and nontone listeners did not differ, *t*(66) = 0.22, *p* = .83.

Accordingly, we planned a second study to guide listeners explicitly to focus on melody changes in one set of trials, and instrument changes on another set of trials. This should allow us to rule out task interpretation as a confound. If listeners in Experiment 1 performed differently due to differing task interpretations, then Akan listeners’ melody change detection should increase and other listeners’ instrument change detection should also increase. However, if Experiment 1 instead reflects true perceptual proclivities, then results should be much the same between the two studies.

## Experiment 2

This second experiment redesigned the task to make the intended responses clearer. First, we divided the task into two short blocks, one with instrument-change trials, the other with melody-change trials, so that listeners did not have to decide to attend to one set of changes at the expense of another. Second, each block was preceded by 12 training trials with feedback, and if accuracy on this training set was below 11/12 correct responses (91.6%), it was repeated. This aimed to clarify for participants the types of changes that they were to listen for.

### Method

#### Participants

We planned to compare Akan speakers across experiments, and to compare all three groups in the current experiment. To detect an interaction in a cross-experiment ANOVA comparing Akan speakers, which would indicate that melody detection relatively improved, assuming a moderate effect size of *f* = .25 and (conservatively) a correlation of 0 for within-subjects measures, we needed 33 participants per group to achieve power ≥.80. To detect an interaction in a Group × Trial Type analysis, again assuming *f* = .25 and correlation = 0, we needed 27 participants per group to achieve power ≥.80. Thus, to parallel Experiment 1 and maintain power, we retained the goal of 40 participants per group.

We tested 41 new Akan-speaking participants from the same pool as before. Dialects reported were Asante Twi (34), and one each Akuapem, Akyem, Leteh, Okwahu, one both Akyem and Akuapem, one both Akyem and Asante Twi, and one both Asante Twi and Akuapem. All reported speaking English as well. At a later time point—delayed due to the onset of Covid-19—we tested two groups of students from the UCSD pool, those who do not speak a tone language and those who do (assuming, correctly, that tone languages would be those of East Asia). To obtain 40 participants in each group in a single run in an online testing system (FindingFive; www.findingfive.com) without having to iteratively eliminate and replace ineligible participants, we tested a total of 64 per group. Then, prior to looking at the data, we selected the first 40 of each group who actually met the specified language criteria and who reported that they took part in a relatively undisturbed auditory environment. This entailed removing 12 participants from the consecutive series. Ten participants from the nontone group were removed due to speaking a tone language or a pitch-accent language, as in Experiment 1; two from the nontone group were removed for reporting auditory disturbances. One of the excluded nontone participants also took part as a tone participant, and their data were removed from that condition as well. The remaining (extra) participants’ data (*n* = 12 nontone and *n* = 23 tone) were not analyzed. The final set of nontone participants reported dominance in English (31), both English and Spanish (5), Spanish (2), both English and Tagalog (1), or all three of Russian, Hebrew, and English (1). Of English-dominant participants, two reported no knowledge of other languages, but the rest reported knowledge of Spanish (22), Tagalog (3), French (2), Hebrew (1), and Kannada (1). The final set of tone participants reported speaking one or more tone languages including Mandarin (23), Chinese (3; presumably Mandarin; these participants did not respond to our request to be more specific than the term “Chinese”), Vietnamese (7), Cantonese (6), or both Taishanese and Cantonese (1).

#### Stimuli

For variety, we developed a second set of 12 melodies and single-note changes using the same set of constraints as before. Due to a counterbalancing oversight, these new melodies occurred only in the different-melody condition rather than counterbalancing across different-melody and different-instrument conditions. The original 12 melodies occurred in the different-instrument condition only. As this pattern was consistent across all participants and groups, this should not matter for group comparisons.

#### Procedure

Akan participants completed the study in person using PsychoPy2 as before, with Akan instructions and responses, while the remaining participants completed the study with English instructions and responses via an on-line experimental presentation system, FindingFive (www.findingfive.com). Akan participants also took part in an unrelated speech perception study, half before and half after the current study.

Each participant completed two blocks of trials: a melody-change block and an instrument-change block. Each block of each type could be one of four prerandomized lists of stimuli, with a corresponding list out of four for the other block (that is, if a participant completed Melody-Change List 1, they also completed Instrument-Change List 1). Block order was counterbalanced across participants and list numbers.

Within a block, participants received 12 training trials, half same, half different. Training trials were designed to alert listeners to the critical differences to listen to in each block. For different-instrument training trials, the first different trial contained a large instrument difference (muted trumpet vs. vibraphone) so that the most obvious different example occurred first. The second different trial contained a smaller instrument difference (bassoon vs. saxophone). The remaining four different trials were divided evenly between large and small instrument differences. For different-melody training trials, of the six “different” examples, the first one differed in contour and in four notes, the second differed in contour and three notes, and the remaining four had matching contours but differed in two notes. The two-note differences were designed to be slightly easier than the main test trials (matching contour, difference of one note) but reasonably challenging so that listeners knew what they were expected to listen for. If these training trials were below 91.6% accuracy (11/12 or more correct), the training sub-block was repeated once. After two passes through the training sub-block, even if the listener did not reach criterion on the training trials, they continued to the main block which contained 48 trials (half same, half different). Thus, the maximum total number of trials an individual participant might complete was (12 × 2 + 48 + 12 × 2 + 48 =) 144.

Not all participants reached criterion within two blocks of training, mostly in the melody task. In the second block of melody training, 21 Akan participants, 14 English participants, and five East Asian tone participants did not reach criterion. On the instrument task, the numbers were six Akan participants, four English, and two East Asian tone. However, almost all of these participants showed performance numerically above chance (.50) in the second block of training. This hints that melodic difference detection in particular is quite challenging, despite understanding of the task itself. In fact, in the second block of training, detection rates on the first two different melody trials, which were distinctly different (3-4 notes differed, contour changes), were .88, but for the latter four different trials (mildly different: 2 notes differed, no contour changes) were only .52 (note that this still exceeded the baseline false-alarm rate, saying “different” for two identical melodies, which was .13). Still, very low training performance by a given participant raises the possibility that that participant did not correctly understand the task. To balance these competing concerns, we excluded data from 6 participants (2 Akan, 3 English, 1 East Asian) who scored below 60% on *either* second training block (melody or instrument) as a precaution. Scores were converted to *d′* with the same adjustment for 0 and 1 values as in Experiment 1.

### Results

As planned, we first compared the current group of Akan-speaking participants to those in Experiment 1, to assess whether pitch change detection improved with clearer instructions. At first glance, the current Akan speakers show similar performance to Akan-speaking participants in Experiment 1: instrument trials showed high detection rates (*d′* = 3.33, *SD* = 0.90), while melody trials showed low detection rates (*d′* = 0.75, *SD* = 0.61), a significant difference, *t*(40) = 18.26, *p* < .0001. We then conducted an ANOVA comparing trial type (within subjects) across experiments (between groups). There was an effect of trial type, *F*(1, 80) = 380.57, *p* < .0001, with higher detection scores for instrument trials than melody trials. An overall effect of experiment, *F*(1, 80) = 5.06, *p* = .03, suggested that clarifying the instructions, providing more examples with feedback, or both led to better overall detection. However, there was no interaction, *F*(1, 80) = 1.31, *p* = .26, suggesting that Akan speakers did not change their response patterns in a way that *selectively* improved melody change detection.

**Fig. 4:**
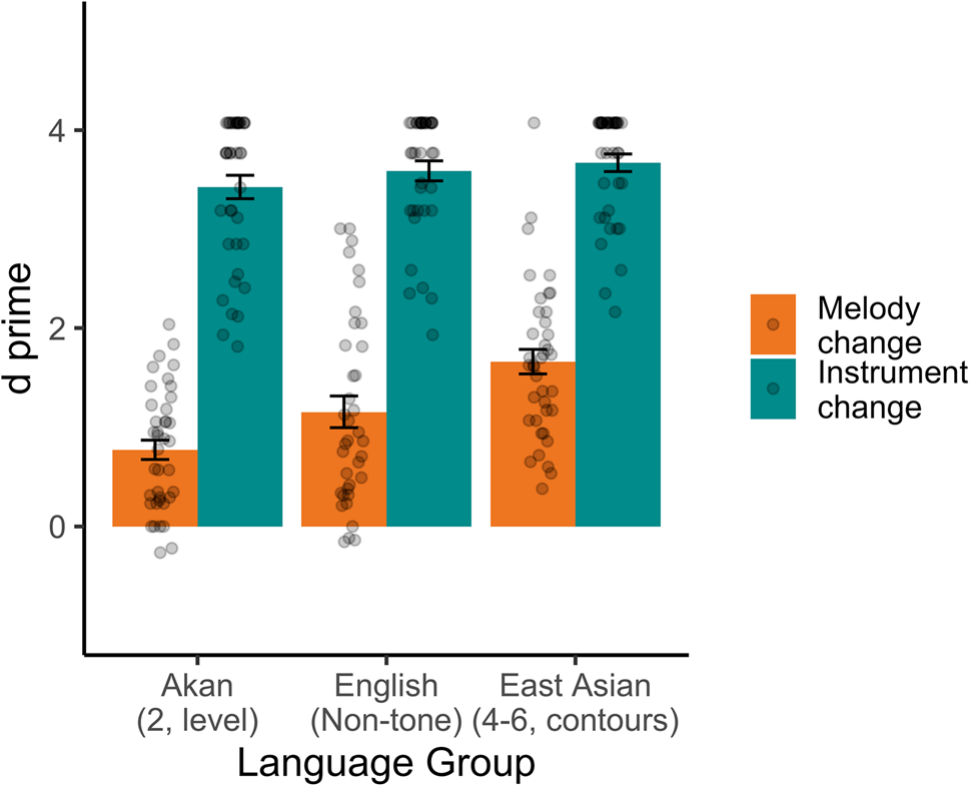
Experiment 2, *d*-primes to melody change vs. instrument change trials across language groups, with standard errors. Points are individual participants

Next, we examined all three groups in Experiment 2 (Fig. 4), subjecting *d′* to a mixed-design ANOVA, with trial type (melody, instrument; within-subjects) and language group (Akan tone, English, East Asian tone; between groups) as factors. Language group was significant, *F*(1, 112) = 9.05, *p* = .0002, with higher *d′* overall for East Asian tone speakers than Akan speakers, *t*(76) = 3.10, *p* = .003, but no differences between East Asian and English, *t*(74) = 1.42, *p* = .16, or Akan and English, *t*(74) = 1.57, *p* = .12. Trial type was also significant, *F*(1,112) = 927.01, *p* < .0001, with higher *d′* overall for instrument-change trials than for melody-change trials. Finally, there was a Language Group × Trial Type interaction, *F*(2, 112) = 5.97, *p* = .003. While all groups showed higher instrument *d′* than melody *d′—*Akan: *t*(38) = 21.23, *p*_*B*_ < .0001; English: *t*(36) = 16.51, *p*_*B*_ < .0001; East Asian: *t*(38) = 15.30, *p*_*B*_ < .0001—the magnitude of this difference varied across groups (difference scores for Akan: 2.65, *SD* = .78; English: 2.43, *SD* = .90; East Asian: 2.01, *SD* = .82). To further understand the nature of these differences, we compared each pair of groups in a 2 × 2 ANOVA. The ANOVA including Akan and English speakers showed no interaction, *F*(1, 74) = 1.28, *p*_*B*_ = .78, suggesting that these two groups had indistinguishable melody performance relative to instrument performance. The East-Asian/English comparison missed significance after Bonferroni correction, *F*(1, 74) = 4.61, *p*_*B*_ = .11. The East Asian/Akan comparison, *F*(1, 76) = 12.50, *p*_*B*_ = .002, did show a significant interaction, indicating that the East Asian group showed a smaller difference between melody and instrument *d′* than Akan speakers. Thus, while the pattern of results appears different from Experiment 1, the East Asian melody advantage over English speakers is numerically present, and the Akan lack of melody advantage replicates.

#### Exploratory: Results taking music experience into account

We asked whether groups differed in music experience by computing an ANOVA on years of music experience, with language group as a predictor. Language group was significant, *F*(2, 111) = 4.38, *p* = .01. Akan speakers showed the fewest years of experience (*M* = 4.0, *SD* = 6.4), English speakers in between (*M* = 6.5, *SD* = 6.8), and East Asian tone speakers the most (*M* = 8.3, *SD* = 6.2). Only Akan and East Asian groups differed significantly in music experience, *t*(75) = 3.03, *p* = .003.

**Fig. 5:**
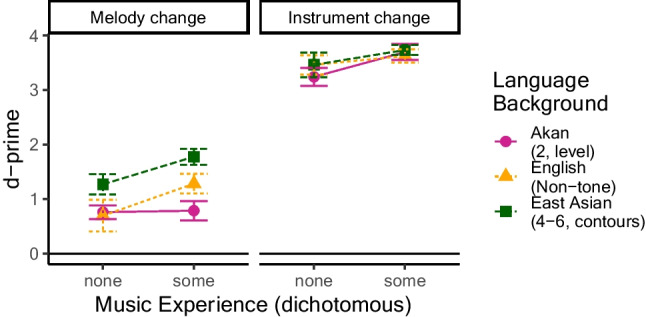
Experiment 2, *d*-prime values, split by absence versus presence of music experience, with standard errors. Melody change detection is stronger with musical experience, but music effects do not explain the effects of language group

This raises the possibility that differences in music experience, rather than language background, dictated results. Results were recomputed with the additional factor of music experience (Fig. 5). Given the large number of “0” values, it was converted to a binary no experience (0 years) versus experience (1+ years) factor. The ANOVA showed an effect of music experience, *F*(1, 108) = 6.98, *p* = .009, with music experience conferring higher *d′* values. Like the original analysis, it showed effects of language group, *F*(2, 108) = 5.43, *p* = .006, trial type, *F*(1, 108) = 921.08, *p* < .0001, and a Language Group × Trial Type interaction, *F*(2, 108) = 5.12, *p* = .007. However, music experience did not interact with any factors—language group: *F*(2, 108) = 0.16, *p* = .85; trial type: *F*(1, 108) = 0.02, *p* = .90; Language Group × Trial Type × Music Experience: *F*(2, 108) = 2.25, *p* = .11. This is not the expected pattern if music experience rather than language group controls the outcome.

#### Exploratory analysis: General attention/carefulness

Another way to equate listeners across groups is to match for scores on the instrument change detection control task. Presumably, listeners who are more attentive will have higher *d′* values in this task. If we look at the subset of listeners who made no errors on the instrument change detection task (Table 3), *d′* scores on the melody change detection task still show numeric differences in the same direction as the full dataset, especially between the two tone language groups.

**Table 3:** Melody scores for listeners with perfect accuracy on instrument change detection

Language group	*d′*	*SD*	*n*
Akan tone	1.04	0.55	15
English nontone	1.62	0.89	17
East Asian tone	1.88	0.68	21

Of course, it is evident that the instrument task is easier than the melody task: 53 participants performed perfectly on the instrument task, but only one on the melody task. One counterexplanation is that, rather than language shaping pitch perception, there is some sort of attentional ceiling that dictates performance generally, and this attentional ceiling (a) differs between groups and (b) has stronger effects on the more-difficult melody task. To examine this, one might look at participants with *below*-ceiling instrument *d′* (say, under 3.5, which means two or more errors) to determine whether melody task accuracy still differs between groups. It does (see Table 4), in the same way as the overall analysis.

**Table 4:** Melody scores for listeners with below-ceiling accuracy on instrument change detection (2+ errors)

Language group	*d′*	*SD*	*n*
Akan tone	0.60	0.59	16
English nontone	0.69	0.80	14
East Asian tone	1.38	0.39	13

### Discussion

This experiment asked whether melodic perception advantages would appear in Akan tone language listeners if the task were made clearer, using clearer instructions, more example trials, and separate blocks for melody and instrument change detection. In short, no. Melody discrimination did not appear to pattern differently than Experiment 1: Akan speakers still showed much higher sensitivity in instrument change detection than melody change detection, while speakers of East Asian tone languages still demonstrated the highest *d′* for melody change detection. The major qualitative difference compared with Experiment 1 is that the English speakers and East Asian tone speakers were more responsive to instrument changes. This might indicate that these two groups interpreted task instructions differently than Akan speakers in Experiment 1. It is possible, though we think unlikely, that including two different types of trials (instrument and melody change) made listeners in Experiment 1 choose one dimension to attend to versus another, with some in the U.S. groups tuning out instrument changes in favor of melody changes, though there is not a ready explanation for why this might happen.

## General discussion

Counter to the tone language hypothesis, there appears to be no tone language advantage for musical pitch perception in speakers of Akan, a two-tone West African language, compared with nontone language (English) speakers. Excellent performance in instrument change detection casts doubt on the counterexplanation that Akan-speaking listeners detect melody changes only modestly because they are simply less familiar with the task itself.

Nonetheless, in groups of participants at a US university, we replicated the previously reported melody change detection advantage for East Asian tone language speakers over nontone language (English) speakers (e.g., Bidelman et al., 2013; Pfordresher & Brown, 2009). That is, in Experiment 1, speakers of East Asian tone languages showed better detection of subtle pitch changes in melodies compared with largely English-dominant nontone language speakers. If one is willing to forgo Bonferroni correction, this advantage compared with English speakers appeared in Experiment 2 as well. This suggests that our research design was adequate to capture previously observed tone language advantages. The open question is why Akan speakers did not show a melody advantage.

### Theoretical implications

Why did we not find a tone-language advantage in Akan speakers, given that Akan is a tone language? Several explanations come to mind, but these explanations vary in their plausibility. The most interesting possible explanation is that effects of speaking a tone language on nonspeech pitch perception vary with how tone is used in a particular language. In particular, multiple tone properties of the East Asian languages in this study might confer greater pitch-processing acuity than tone properties of Akan. First, the number of contrastive tones in Akan is two,^2^ although there are more surface pitch levels due to the phenomenon of “downstep,” which lowers high tones successively in a sequence. In contrast, the East Asian tone languages in our sample contained four contrastive tones or more. Second, the type of tones are different. Akan is a level tone language whereas the tones in the other languages tested here are predominantly contours, which involve pitch slope and pitch alignment differences within the vowel or syllable. It is possible that representing contour tones is critical to pitch perception advantages because tones that change over time provide a stronger framework to “chunk” longer melodies more concisely (i.e., to store them more compactly in memory). For example, a falling sequence of musical pitches could be mapped to a single falling tone in a contour tone language, but to two level tones in a level tone language. It is also possible that contour tones are more complex to represent because they contain dynamic rather than static pitch information, and thus lead to a more precise encoding of pitch information.

Third, functional load of tone (how much “work” it does in distinguishing words from each other) may differ between East Asian tone languages and African two-tone languages like Akan. The functional load of tone in Mandarin has been reported to be equivalent to that of vowels (Surendran & Levow, 2004). While it is difficult to determine functional load of tone in Akan without corpora, it might be lower than in the East Asian tone languages in the study, meaning that tone misperception would impact comprehension less strongly. Relatedly, it is the case that tone is used grammatically in Akan to express tense-aspect-mood distinctions. This means that many single lexemes (words) do not have consistent tone, as it changes in grammatical contexts. For bisyllabic or longer words, this means there is a tone melody imposed on the word, such as high-low or low-high, requiring attention to a more global pitch contour than that expressed on a single syllable. While some East Asian languages have tone sandhi effects—that is, cases where the tone(s) of a word changes in context—this is nevertheless minimal in Mandarin (Chen, 2000), the most commonly occurring tone language spoken by our East Asian tone participants. Cantonese (Bauer & Benedict 1997; Zhang, 2014) and Vietnamese (Kirby, 2011) also have little tone sandhi. The hypothesis that Akan facilitates more global contour features rather than fine-grained pitch changes was not tested in the current task, but could be assessed in future work.

A second explanation for the lack of tone advantages in Akan speakers is that Ghanaian listeners are familiar with different musical systems compared with English and East Asian tone language speakers, and they performed less well at detecting melody changes because the melodies did not match their internalized tonal representations as well. There are indigenous Ghanaian musical forms that do not use the Western major scale that melodies were loosely based on, but many of our participants in Ghana reported listening to or participating in musical styles related to Western music. Experiment 2 included questions on musical styles listened to: among Akan listeners, 90% reported listening to Gospel music; 68% to Highlife music, 32% to Hip-Hop, 37% to Hiplife, and 24% to Reggae. The Highlife musical style originated with Ghanaian musicians who blended traditional Akan music with Western instruments. Many of those Western instruments physically impose a 12-pitch tuning system (a superset of the major scale that allows 12 different major scales to be played), though this does not necessarily entail that Ghanaian musicians use these pitches in the same way (see Agawu, 2016, for interesting discussion of the effects of colonization and Western tonality on various musical forms in African nations, with particular focus on Ghana). Further, some studies suggest that listeners exposed to two different musical systems can discover rhythmic (Hannon & Trehub, 2005) and tonal (Matsunaga et al., 2020) properties of both systems by adulthood. Thus, we think it is unlikely that our Akan-speaking listeners were less familiar with the Western pitch collections used in the study. Still, it is possible that Akan speakers might excel at detecting changes to melodies more closely based on musical styles familiar to them (Castellano et al., 1984; Krumhansl et al., 1999). A cross-cultural study in which Akan speakers showed no advantage in pitch change detection even for more-familiar tone sets would make an even more convincing case for lack of tone language advantage in Akan speakers. We believe this is a fruitful direction for future work in conjunction with knowledgeable musician collaborators.

A final possibility is that the apparent lack of Akan tone language advantage represents some correlate of differences in the type of educational enculturation amongst the tested populations with regard to test-taking, which might obscure tone language benefits. Nontone speakers in the U.S. have extensive experience with Americanized test-taking practices, similar to the experimental procedures used here, as do East Asian tone language speakers who have moved to the U.S. Further, students in or from China (who made up the majority of our tone-language samples here) have perhaps even more intensive practice than English-speaking U.S. students in preparation for the *gaokao* exams (Larmer, 2014). Akan speakers in Ghana have less exposure to this testing style. While the more extensive training trials in Experiment 2 aimed to familiarize all participants with the testing procedure we used, it is not likely that a few minutes of training have as much effect as hundreds of hours of practice with multiple-choice tests over many years. One previous finding speaks against this explanation: speakers of Yoruba, who were in part a community-based sample (Nigerian immigrants to the U.S.) and thus not particularly likely to have experience with U.S. test-taking practices, nevertheless show a tone-language advantage over English speakers (Bradley, 2016).

In conclusion, our study shows that conclusions about tone languages conferring pitch processing advantages in music need to be nuanced. Such conclusions may hold for only a subset of tone languages, perhaps those with a larger number of tones than two, and may be influenced by how tone functions in a given language. Indeed, the Akan speakers in our study did not show the pitch melody detection ability that Yoruba speakers displayed in Bradley (2016), suggesting that even somewhat related languages can have different effects on pitch perception. Further studies should incorporate additional African tone languages with different tone inventories and systems, as well as tone languages from other areas of the world, including the Americas.

## Footnotes

1 Subscript numerals refer to the approximate pitch height of particular notes, with lower numbers referring to lower pitches. C_4_ is middle C; A_3_ is the A below middle C, E_4_ is the E above middle C, and so forth.

2 This is comparable to the number of pitch contrasts in so-called pitch-accent languages (as diverse as Japanese, Croatian, and Swedish), which may show slight musical pitch advantages compared with nontone languages (e.g., Liu et al., 2021). It is an open question how similar pitch use is across these otherwise disparate languages, and how similar they are to two-tone level systems as in Akan (see Hyman, 2009, for discussion of difficulty in classifying pitch-accent languages as distinct from tone languages).
